# Syntheses and Characterization of Novel Perovskite-Type LaScO_3_-Based Lithium Ionic Conductors

**DOI:** 10.3390/molecules26020299

**Published:** 2021-01-08

**Authors:** Guowei Zhao, Kota Suzuki, Masaaki Hirayama, Ryoji Kanno

**Affiliations:** 1 All-Solid-State Battery Unit, Institute of Innovation Research, Tokyo Institute of Technology, 4259 Nagatsuta, Midori-ku, Yokohama 226-8502, Japan; zhao.g.w@echem.titech.ac.jp; 2 Department of Chemical Science and Engineering, School of Materials and Chemical Technology, Tokyo Institute of Technology, 4259 Nagatsuta, Midori-ku, Yokohama 226-8502, Japan; suzuki.k.bf@m.titech.ac.jp (K.S.); hirayama@echem.titech.ac.jp (M.H.); 3 Precursory Research for Embryonic Science and Technology (PRESTO), Japan Science and Technology Agency (JST), 4-1-8 Honcho, Kawaguchi-shi, Saitama 332-0012, Japan

**Keywords:** lithium ionic conductor, perovskite structure, solid electrolyte, oxide

## Abstract

Perovskite-type lithium ionic conductors were explored in the (Li*_x_*La_1−*x*/3_)ScO_3_ system following their syntheses via a high-pressure solid-state reaction. Phase identification indicated that a solid solution with a perovskite-type structure was formed in the range 0 ≤ *x* < 0.6. When *x* = 0.45, (Li_0.45_La_0.85_)ScO_3_ exhibited the highest ionic conductivity and a low activation energy. Increasing the loading of lithium as an ionic diffusion carrier expanded the unit cell volume and contributed to the higher ionic conductivity and lower activation energy. Cations with higher oxidation numbers were introduced into the *A*/*B* sites to improve the ionic conductivity. Ce^4+^ and Zr^4+^ or Nb^5+^ dopants partially substituted the *A*-site (La/Li) and *B*-site Sc, respectively. Although *B*-site doping produced a lower ionic conductivity, *A*-site Ce^4+^ doping improved the conductive properties. A perovskite-type single phase was obtained for (Li_0.45_La_0.78_Ce_0.05_)ScO_3_ upon Ce^4+^ doping, providing a higher ionic conductivity than (Li_0.45_La_0.85_)ScO_3_. Compositional analysis and crystal-structure refinement of (Li_0.45_La_0.85_)ScO_3_ and (Li_0.45_La_0.78_Ce_0.05_)ScO_3_ revealed increased lithium contents and expansion of the unit cell upon Ce^4+^ co-doping. The highest ionic conductivity of 1.1 × 10^−3^ S cm^−1^ at 623 K was confirmed for (Li_0.4_Ce_0.15_La_0.67_)ScO_3_, which is more than one order of magnitude higher than that of the (Li*_x_*La_1−*x*/3_)ScO_3_ system.

## 1. Introduction

All-solid-state lithium batteries have recently received considerable attention as safer, stable, compact, and reliable energy storage devices [1,2]. However, suitable lithium-based solid electrolytes are required for their fabrication [3,4,5,6]. In the past few decades, a range of crystalline and amorphous materials have been examined to prepare solid electrolytes [7,8,9], which can be roughly divided into two groups, namely sulfides [1,10,11,12] and oxides [13,14,15,16]. However, many of the sulfide-based compounds are unstable under air and react with the Li metal electrode [17]. In contrast, the oxide-based group is more stable and easily prepared. As a result, the latter is relatively convenient for battery fabrication in a dry air atmosphere [6].

In the oxide group, Li-La-Ti-O perovskite [13,18,19,20,21] is considered a promising candidate for use as a battery electrolyte due to its high ionic conductivity at room temperature (>10^−3^ S cm^−1^) [13]. However, high temperatures (>1000 °C) are required for its preparation and it suffers from electrochemical stability issues due to the reduction of the reducible element Ti^4+^ to Ti^3+^ during the electrochemical process, which gives rise to undesirable electronic conduction properties and ultimately affects its practical application [20]. Although lithium ionic conductors developed in this perovskite system have mainly been prepared based on the introduction of lithium vacancies at the *A*-site [13,22], the development of lithium ionic conductors through the introduction of lithium interstitials in the perovskite-type system has generally not been considered.

We herein focus on LaScO_3_ [23] as a mother structure for solid electrolytes to investigate the novel perovskite system. We previously considered that the *B* site cation of Sc^3+^ would be expected to show a higher resistance to electrochemical reduction than Ti^4+^ [24]. In addition, it was thought that the low valent Li^+^ cation could partially substitute the high valent La^3+^ cation, which could result in the generation of structural defects due to charge compensation, with examples including lithium-ion interstitials that may act as ion carriers for ion diffusion. Therefore, we herein report the development of lithium ionic conductors in the LaScO_3_-based perovskite system. Initially, different ratios of lithium are introduced into LaScO_3_ according to the chemical formula (Li*_x_*La_1−*x*/3_)ScO_3_, and their ionic conductivities are evaluated. To improve the ionic conductivity of (Li*_x_*La_1−*x*/3_)ScO_3_, further modification is carried out by introducing additional structural defects. As the dopant, Ce^4+^ and Zr^4+^ or Nb^5+^ are selected to partially substitute the *A*- or *B*- sites of (Li*_x_*La_1−*x*/3_)ScO_3_, and perovskite-type lithium-excess lithium ionic conductors are developed in a step-by-step manner. Finally, the composition exhibiting the highest conductivity is identified in the prepared LaScO_3_ perovskite-based materials.

## 2. Results and Discussion

### 2.1. Syntheses and Ionic Conductivities of (Li_x_La_1−x/3_)ScO_3_

**Figure 1:**
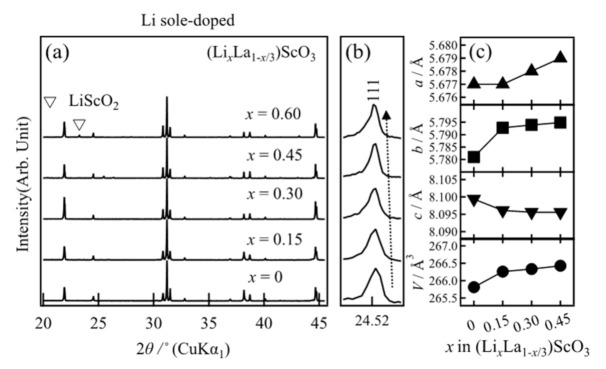
(**a**,**b**) X-ray diffraction patterns and (**c**) dependence of the lattice parameters on the composition of (Li_x_La_1−x/3_)ScO_3_ (*x* = 0, 0.15, 0.30, 0.45). Lattice parameter data for LaScO_3_ (*x* = 0) were taken from the literature [25].

The X-ray diffraction (XRD) patterns of (Li*_x_*La_1−*x*/3_)ScO_3_ (*x* = 0–0.60) are shown in Figure 1a,b. The diffraction peaks of the main phase were identified to correspond to the orthorhombic *Pnma* LaScO_3_ phase [25]. For the composition where *x* = 0.6, additional peaks attributed to the impurity (LiScO_2_) phase were also observed, indicating that the solid solution limit with the LaScO_3_ structure is *x* < 0.6. The peak corresponding to the 111 diffraction peak at ~24.5° did not show a clear shift upon increasing the *x* value (i.e., the Li^+^ content), although the lattice volume increased slightly (Figure 1c). This indicates that a solid solution formed through lithium doping in the *x* range of 0.15–0.45. The absence of a significant diffraction peak shifts may be attributed to the introduced lithium ions located at the interstitial positions, which are not sufficient to affect the lattice change. The total content of La and Li at *A*-site exceeds 1 when *x* > 0, which indicates that a portion of the lithium ions may be located at the interstitial sites in addition to the *A*-sites within the structure of LaScO_3_. These results clearly indicate that lithium-excess perovskite-type materials were successfully formed via this high-pressure synthesis route.

**Figure 2:**
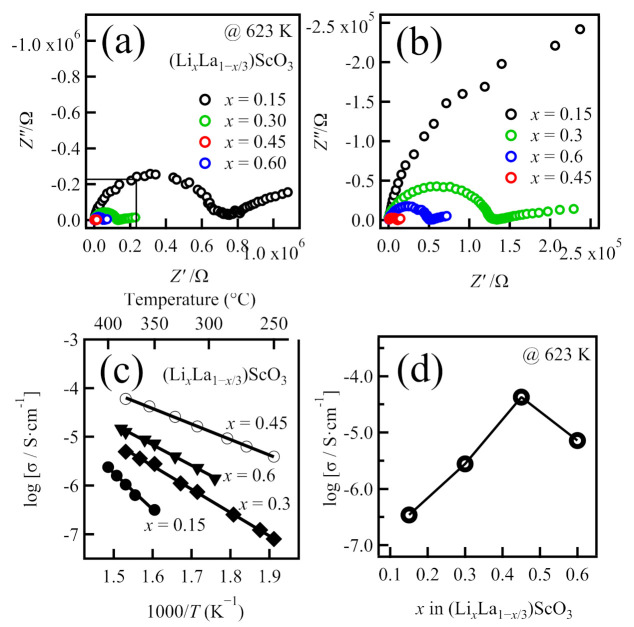
(**a**,**b**) Impedance plots obtained at 623 K, (**c**) temperature dependence of the conductivity, and (**d**) variation in the conductivity at 623 K based on the composition of (Li*_x_*La_1−*x*/3_)ScO_3_ (*x* = 0.15, 0.30, 0.45, 0.60).

To determine the ionic conductivities of the samples, alternating current (a.c.) impedance measurements were conducted. The total resistance, *R*, was determined by calculating the intercept value of the semicircular plot with the *x*-axis. The ionic conductivities were calculated using σ = *d*/(*R* × *A*), where *R* is the resistance, *d* is the thickness, and *A* is the area of the pellet. It should be noted that the a.c. impedance could not be measured for the samples at temperatures < 523 K. Therefore, to compare these ionic conductivities with the actual measured experimental data, the ionic conductivity obtained at the intermediate temperature (623 K) of the test temperature interval was chosen. Figure 2a,b show the typical complex impedance spectra of all sintered compounds at 623 K. These spectra are composed of a semicircle at higher frequencies and a spike at lower frequencies, which correspond to the contributions of the bulk/grain boundary and electrode resistances, respectively. A semicircle with a capacitance value on the order of 10^−11^ F indicates a total resistance that consists of a mixed contribution (sum of the bulk and grain boundaries). Since the component separation for the bulk and the grain boundary resistance could not be obtained, the total conductivity was calculated from the semicircle as a total resistance.

Figure 2c shows the temperature dependences of the conductivities, while Figure 2d displays the conductivity at 623 K as a function of the composition (*x*). Table 1 summarizes the ionic conductivities measured at 623 K and the activation energies *E*_a_ for the compounds. As indicated, the conductivity continuously increased upon increasing *x* from 0 to 0.45, and then slightly decreased where *x* > 0.45. The highest conductivity was observed for the composition with *x* = 0.45, yielding a value of 4.2 × 10^−5^ S cm^−1^ at 623 K with a low activation energy of 61 kJ mol^−1^. The decrease in conductivity for the sample where *x* = 0.60 was likely due to the formation of the impurity phase LiScO_2_, which exhibits a relatively low ionic conductivity at the same temperature (~10^−9^ S cm^−1^ at 623 K) [26]. As the sample where *x* = 0.45 exhibits the highest ionic conductivity, (Li_0.45_La_0.85_)ScO_3_ was determined to be the optimal initial composition for examining the co-doping systems containing Ce^4+^, Zr^4+^, and Nb^5+^.

### 2.2. Syntheses and Ionic Conductivities of (Li_x_La_1−x/3_)ScO_3_ co-doped with Ce^4+^, Zr^4+^, and Nb^5+^

**Figure 3:**
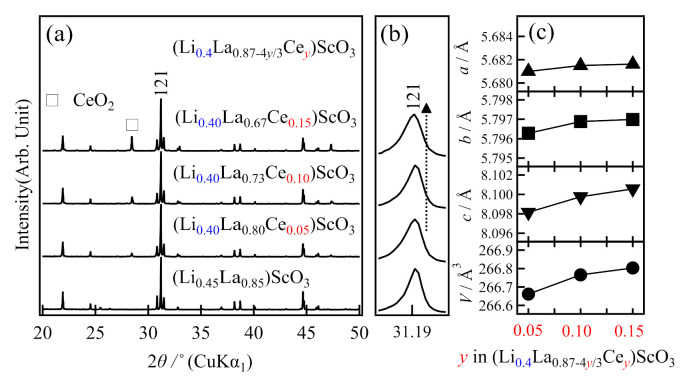
Variation in (**a**,**b**) the X-ray diffraction patterns, and (**c**) the lattice parameters based on the composition of the as-prepared (Li_0.4_La_0.87−4*y*/3_Ce*_y_*)ScO_3_ (*y* = 0.05, 0.10, 0.15).

For the *A*-site doping system, two material search directions were examined. The first was La^3+^ substitution by Ce^4+^ with fixing of the lithium composition according to the formula (Li*_x_*La_0.87−4*y*/3_Ce*_y_*)ScO_3_, where *x* = 0.35 or 0.40. The XRD patterns of the obtained samples are shown in Figure 3. Although the LaScO_3_ phase accounted for the main diffraction peaks, an additional peak derived from the CeO_2_ impurity was observed at ~28°. The intensity of this peak increased upon increasing the *y* value (i.e., the amount of Ce doping), while the lattice parameters increased slightly at the same time, as shown in Figure 3c. These results indicate that compositional and/or structural changes occurred depending on the *y* value, although the mono-phasic perovskite-type solid solution was not obtained.

**Figure 4:**
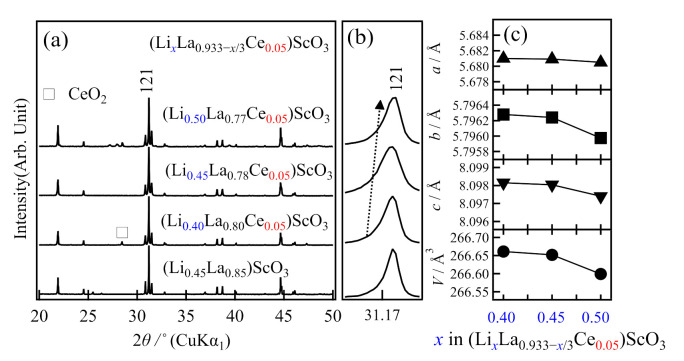
Variation in (**a**,**b**) the X-ray diffraction patterns, and (**c**) the lattice parameters based on the composition of the as-prepared (LixLa0.933−x/3Ce0.05)ScO3 (x = 0.4, 0.45, and 0.50).

The second direction of *A*-site doping was conducted using a fixed Ce content. In this case, charge neutrality was maintained using the La:Li ratio, giving (Li*_x_*La_0.933−*x*/3_Ce*_y_*)ScO_3_, where *y* = 0.05 was the target composition. The XRD patterns of the obtained products are shown in Figure 4. The sample of (Li_0.45_La_0.78_Ce_0.05_)ScO_3_ where *x* = 0.45 exhibited the mono-phasic characteristic of the LaScO_3_ perovskite, while other compositions showed impurity formation. Even in this case, the small changes in the lattice parameters, which indicate compositional and/or structural changes, were confirmed (Figure 4c).

*B*-site doping using Zr^4+^ or Nb^5+^ was subsequently examined according to the formulae (Li_0.45_La_0.85−*z*/3_)(Sc_1−*z*_Zr*_z_*)O_3_ and (Li_0.45_La_0.85−2*z*/3_)(Sc_1−*z*_Nb*_z_*)O_3_. As shown in Figures S1 and S2, evident impurity (LiLaO_2_) formation was confirmed even with a small amount of doping (*z* = 0.05) for both dopants. Therefore, *B*-site doping reduces the lithium content in the perovskite-phase due to part of the lithium source being consumed by the impurity. As indicated in Table 2, where the obtained phases of all doping systems are summarized, the synthetic conditions employed herein did not result in a wide range of single-phase solid solutions upon *A*- or *B*- site co-doping into the (Li, La)ScO_3_ system. As a result, (Li_0.45_La_0.78_Ce_0.05_)ScO_3_ was found to be the singular example of a pure LaScO_3_-type phase considering all examined compositions.

**Figure 5:**
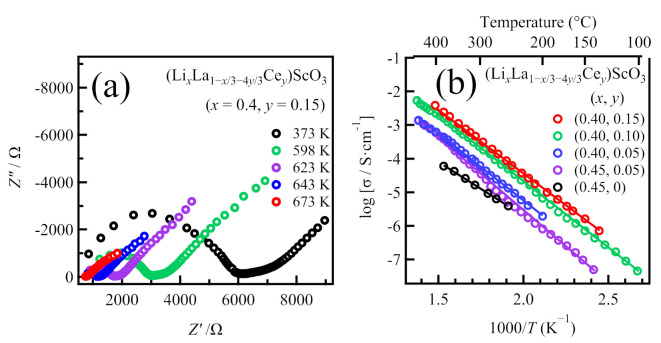
(**a**) Impedance plots of the representative co-doped (Li*_x_*La_1−*x*/3_)ScO_3_ at various temperatures, and (**b**) temperature dependence of the conductivity of the representative samples in (Li*_x_*La_1−*x*/3−4*y*/3_Ce*_y_*)ScO_3_.

All the co-doped samples were then subjected to a.c. impedance measurements; Figure 5a shows the typical a.c. complex impedance spectra of the sample with the nominal composition (Li_0.40_La_0.67_Ce_0.15_)ScO_3_ (*x* = 0.4, *y* = 0.15) recorded at a range of temperatures. The conductivities of the samples were calculated following the same manner as that employed for the non-doped (Li*_x_*La_1−*x*/3_)ScO_3_ system. Thus, Figure 5b presents the Arrhenius plots showing the effect of temperature on the conductivity for the representative samples of (Li*_x_*La_1−*x*/3−4*y*/3_Ce*_y_*)ScO_3_. Since it was not possible to measure the a.c. impedance for every sample at 373 K, the representative conductivities at a higher temperature of 623 K were examined for comparison; the representative conductivities at 623 K and the activation energies for the co-doped samples are summarized in Table 2.

For the Ce^4+^ co-doped system, a number of compositions were found to exhibit higher conductivities than the (Li*_x_*La_1−*x*/3_)ScO_3_ system. For example, (Li_0.45_La_0.78_Ce_0.05_)ScO_3_, which shows single-phase characteristics, presented a relatively high ionic conductivity of 1.9 × 10^−4^ S cm^−1^ at 623 K. This value is nearly five times higher than that of (Li_0.45_La_0.85_)ScO_3_ at the same temperature. The highest ionic conductivity (i.e., 1.1 × 10^−3^ S cm^−1^ at 623 K) was obtained for the Ce^4+^ co-doped (Li_0.4_La_0.67_Ce_0.15_)ScO_3_, even though it contains an impurity. As noted above, a.c. impedance data could be obtained for all the Ce^4+^ co-doped samples at a temperature of 373 K, and below 523 K, it was also not possible to obtain these data for (Li*_x_*La_1−*x*/3_)ScO_3_, which demonstrates that these Ce^4+^ co-doped samples have a higher ionic conductivity than that of (Li*_x_*La_1−*x*/3_)ScO_3_. In contrast, the activation energy was increased upon Ce^4+^ co-doping (e.g., 75.2 kJ mol^−1^ for (Li_0.4_La_0.67_Ce_0.15_)ScO_3_). The enhancement in the ionic conductivity at the same temperature could, therefore, originate from the increase in the pre-exponential factor (σ_0_) in the Arrhenius equation: σ*T* = σ_0_exp(−*E*_a_/*kT*) [5,27]. Since σ_0_ is related to the intrinsic number of carriers and defects, Ce^4+^ co-doping may increase the number of charge carriers, which in turn contributes to ion migration.

The temperature dependences of the conductivities for the (Li_0.45_La_0.85−*z*/3_)(Sc_1−*z*_Zr*_z_*)O_3_, (Li_0.45_La_0.83−1.33*n*_)(Sc_0.95_Zr_0.05_)O_3_, and (Li_0.45_La_0.85−2*z*/3_)(Sc_1−*z*_Nb*_z_*)O_3_ samples are shown in Figure S3 (Supporting Information). More specifically, no improvement in the conductivity was observed for the Zr^4+^ and Nb^5+^ co-doped systems, and relatively high activation energies were confirmed. The lack of an increase in the ionic conductivity may be due to impurities that consume part of the lithium source in the co-doped samples, thereby decreasing the content of mobile lithium ions. The increase observed for the (Li*_x_*La_1−*x*/3_)ScO_3_ system was achieved by *A*-site Ce^4+^ co-doping, while the presence of *M* at the *B*-site was found to have no effect on the conductivity. 

To investigate the electronic contribution to the total conductivity, direct current (d.c.) potentiostatic polarization measurements were performed for (Li_0.4_La_0.67_Ce_0.15_)ScO_3_, which exhibited the highest conductivity of the examined samples. The steady-state current at specific constant voltages of the symmetrical cell Au|(Li_0.4_La_0.67_Ce_0.15_)ScO_3_|Au was evaluated by varying the applied voltage from 0.5 to 1.2 V at 623 K (Figure S4); a rapid current decay was observed shortly following a voltage application. From the slope of the *I* vs. *V* plot, the electronic conductivity of (Li_0.4_La_0.67_Ce_0.15_)ScO_3_ was determined to be 7.6 × 10^−7^ S cm^−1^, indicating that electron transport is negligible in (Li_0.4_La_0.67_Ce_0.15_)ScO_3_ because the calculated value is three orders of magnitude lower than the total conductivity estimated by the a.c. impedance measurements. The conductivity observed for the Ce^4+^-doped (Li_0.4_La_0.67_Ce_0.15_)ScO_3_ was therefore attributed mainly to lithium ion diffusion.

These results therefore indicated that a small amount of Ce^4+^ co-doping enhanced the ionic conductivity. However, the critical factors involved in determining the observed high ionic conductivities remained unclear, and so crystal structure analysis and chemical composition analysis were subsequently carried out.

### 2.3. Crystal Structure Analysis

(Li_0.45_La_0.85_)ScO_3_ (*x* = 0.45) and (Li_0.45_La_0.78_Ce_0.05_)ScO_3_ (*x* = 0.45, *y* = 0.05) were taken as representative highly-conductive samples for the doped and co-doped systems, respectively. These samples were also found to show single-phase characteristics, as discussed previously in the context of XRD phase identification. The crystal structures of these samples were then determined by the Rietveld analysis of synchrotron X-ray diffraction data obtained at 298 K. An orthorhombic perovskite-type structural (LaScO_3_) [25] with *Pnma* symmetry was applied as an initial model for analysis. The atomic positions for the structure refinement models were as follows: La at the 4*c* sites, Sc at the 4*a* sites, O at the 4*a* and 8*d* sites.

During the structural refinement, some Li^+^ was assumed to be present in the *A*-sites (La^3+^), and the occupancy factor of the normal-site lithium (*A*-site) was constrained as 1–*g*(La) for both samples. The lithium present at the interstitial sites was not refined since X-rays are not sensitive to the light element lithium. The lattice constants, atomic isotropic displacement parameters (*B*_iso_), fractional atomic coordinates, atomic occupancies, background parameters, ratio, zero-shift, and profile parameters were then refined. It is worth mentioning that the occupancy of Ce^4+^ was not considered as a refinement parameter because it possesses the same number of electrons as La^3+^, which prevents its identification by X-ray analysis. Therefore, the composition of La and Ce in (Li_0.45_La_0.78_Ce_0.05_)ScO_3_ was estimated by the combination of the La amount obtained by the Rietveld refinement and the La/Ce ratio obtained from inductively coupled plasma atomic emission spectroscopy (ICP-AES) analysis (see Table S1, Supporting Information).

**Figure 6:**
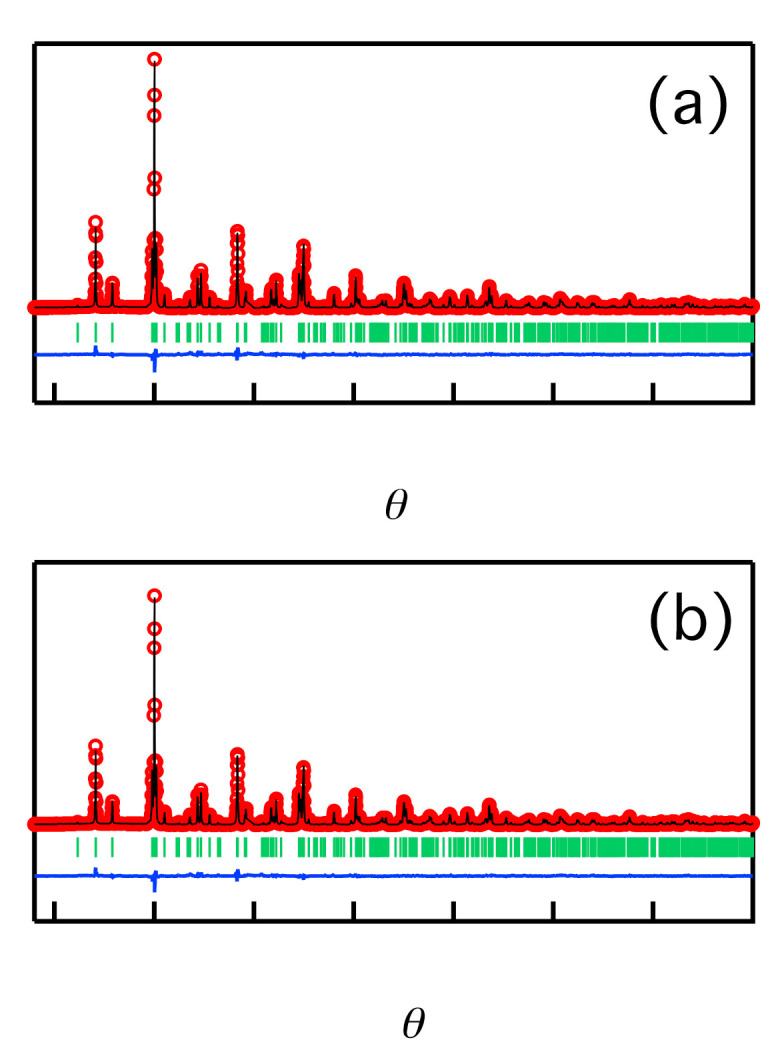
(Color online) Synchrotron Rietveld refinement patterns for (Li_0.45_La_0.85_)ScO_3_ (**a**) and (Li_0.45_La_0.78_Ce_0.05_)ScO_3_ (**b**) at 298 K. Red: observed intensities; black: calculated intensities; and blue: difference plots. Green vertical markers denote the positions of the Bragg reflections of the space group *Pnma* (62) (perovskite structure).

Figure 6 shows the Rietveld refinement patterns, which are also presented in Table 3 and Table 4. All diffraction peaks of the two samples were assigned to the orthorhombic perovskite structure. The obtained reliability factors (*S* and *R*_wp_) confirmed that the proposed structural model for the diffraction data was reasonable.

The refined composition of (Li_0.45_La_0.85_)ScO_3_ was calculated to be Li_0.062_La_0.938_ScO_3_. Subsequently, the interstitial lithium, which cannot be detected by Rietveld analysis, was considered to satisfy the charge neutrality in the sample. Thus, the composition of (Li_0.45_La_0.85_)ScO_3_ was determined to be Li_0.186_La_0.938_ScO_3_, and this value is comparable to that obtained by ICP-AES (Table S1). These analyses revealed that the practical amount of lithium is less than half of the nominal composition of (Li_0.45_La_0.85_)ScO_3_, which was attributed to the reaction of lithium reaction with the platinum capsule during the synthetic procedure [28]. 

The composition of the Ce^4+^ co-doped sample was also determined using the above process. From the Rietveld analysis, Li_0.1788_La_0.8212_ScO_3_ was obtained, whereby the La composition was divided into La and Ce using the La/Ce ratio obtained from the ICP-AES results. Considering the charge neutrality, the composition of Li_0.493_La_0.777_Ce_0.044_ScO_3_ was determined for the co-doped (Li_0.45_La_0.78_Ce_0.05_)ScO_3_. This value is comparable to both the nominal and ICP-AES results (Table S1). 

The significant difference in the lithium amount between the doped and co-doped samples was clarified based on structural and compositional analysis results. More specifically, only 41% of the lithium present in the nominal composition was incorporated into the doped case sample. 

In contrast, a nearly nominal composition was confirmed in the Ce^4+^ co-doped case. Therefore, the observed enhancement in the ionic conductivity upon co-doping was attributed mainly to the abundant lithium (carrier) number in the crystal structure despite a low doping level of 0.05. This assumption is consistent with the change in activation energy observed upon co-doping, which indicated an increase in the number of charge carriers.

The lattice parameters of the compound (Li_0.45_La_0.85_)ScO_3_ were determined to be *a* = 5.67921(5), *b* = 5.79488(4), *c* = 8.09558(6) Å, and *V* = 266.4281(36) Å^3^, while those of (Li_0.45_La_0.78_Ce_0.05_)ScO_3_ were *a* = 5.68088(6) Å, *b* = 5.79624(6) Å, *c* = 8.09803(9) Å, and *V* = 266.6500(48) Å^3^. Compared with LaScO_3_ [25] (*a* = 5.6803, *b* = 5.7907, *c* = 8.0945 Å, and *V* = 266.252 Å^3^), the cell volumes were expanded by +0.07% and +0.15%, respectively. The co-doped (Li_0.45_La_0.78_Ce_0.05_)ScO_3_ presented a larger unit cell size than (Li_0.45_La_0.85_)ScO_3_. Such an expansion of the cell volume may also contribute to the higher ionic conductivity of (Li_0.45_La_0.78_Ce_0.05_)ScO_3_, because a larger unit cell size implies larger channels for lithium ion diffusion.

Again, the highest ionic conductivity (i.e., 1.1 × 10^−3^ S cm^−1^ at 623 K) for the Ce^4+^ co-doped (Li_0.4_La_0.67_Ce_0.15_)ScO_3_ was almost two orders of magnitude higher than that of (Li_0.45_La_0.85_)ScO_3_ at the same temperature. The increase in the lithium content as an ionic diffusion carrier, in addition to the expansion of unit cell volume, was thereby considered to contribute synergistically to the higher ionic conductivity of the Ce^4+^ co-doped system. Determination of the interstitial lithium sites and the La/Ce distribution in the structure through neutron diffraction studies will be conducted in future studies.

## 3. Materials and Methods 

Target materials with the general formula (Li*_x_*La_1−*x*/3_)ScO_3_ and those containing Ce^4+^, Zr^4+^, and Nb^5+^ doping were synthesized via a solid-state reaction using the previously reported high-pressure method [28,29]. The starting materials were La_2_O_3_ (Kanto Chemical Co., Inc., Tokyo, Japan, ≥99.99% purity), Li_2_O_2_ (Kojundo Chemical Laboratory Co. Ltd., Sakado, Saitama Pre., Japan, 99% purity), Sc_2_O_3_ (Alfa Aesar, Thermo Fisher Scientific, Waltham, MA, USA, ≥99.9% purity), CeO_2_ (Wako Pure Chemical Industries, Ltd., Osaka, Japan, ≥99.9% purity), ZrO_2_ (Wako Pure Chemical Industries, Ltd., ≥99.9% purity), and Nb_2_O_5_ (Wako Pure Chemical Industries, Ltd., Osaka, Japan, ≥99.9% purity). Generally, the perovskite system can be synthesized at high temperatures (>1423 K) in an open ambient pressure atmosphere, which often results in lithium evaporation [22]. In this work, the mixed samples were encapsulated in a platinum capsule and subjected to high-pressure synthesis at 2 GPa and 1173–1373 K for 30 min, then quenched by cooling to room temperature. This high-pressure route ensured the incorporation of an accurate amount of Li, in addition to accelerating the reaction.

The synthesized products were characterized by XRD (Rigaku Smart Lab, and Miniflex 300, Rigaku, Tokyo, Japan) using Cu-*K*α radiation under an air atmosphere. Diffraction data were collected using a 0.01° step interval and scanning over the range of 10–50°. The lattice constants were determined using Si (SRM640d) as an internal standard for calibration. Synchrotron XRD patterns were recorded at 298 K using the BL02B2 and BL19B2 beamlines at the SPring-8 facility, operating with a wavelength of 0.5 Å. Structural parameters were refined by the Rietveld method using the RIETAN-FP program [30]. Rietveld analyses were conducted to refine the structural parameters using the diffraction data collected at each 0.01° step width over a 2*θ* range of 10–70°. Chemical analysis was performed by ICP-AES (ICPS-8100, Shimadzu, Tokyo, Japan).

The ionic conductivity of each sample was evaluated by a.c. impedance spectroscopy using a frequency response analyzer (Solartron 1260, AMETEK Scientific Instruments, Berwyn, PA, USA). A gold paste was painted onto each side of the sample as a blocking electrode. The pellets (~3 mm diameter, ~1 mm thickness) were then heated at 300 °C for 30 min under an argon atmosphere to obtain dry samples for carrying out the measurements. The samples were placed under an argon flow, and the measurements were performed between 298 and 673 K at frequencies ranging from 0.1 Hz to 3 MHz with an applied voltage of 20–100 mV. 

The electrical conductivity of each sample was measured using the Hebb-Wagner polarization method [31]. The voltage, which was controlled using a potentiostat electrochemical interface (Solartron 1287, AMETEK Scientific Instruments, Berwyn, PA, USA), was applied from 0.1 to 1.2 V. The resulting current obtained due to the electronic contribution was evaluated.

## 4. Conclusions

Lithium ionic conductors were developed in the (Li*_x_*La_1−*x*/3_)ScO_3_ system with a perovskite structure via a high-pressure solid-state reaction. The resulting novel lithium-excess perovskite-type materials exhibited solid solution formation where 0 ≤ *x* < 0.60. The highest ionic conductivity was observed for (Li_0.45_La_0.85_)ScO_3_, yielding a conductivity of 4.2 × 10^−5^ S cm^−1^ at 623 K with a low activation energy of 61 kJ mol^−1^. Further improvement in the ionic conductivity of (Li_0.45_La_0.85_)ScO_3_ was examined by introducing aliovalent cations; Ce^4+^ and Zr^4+^ or Nb^5+^ were selected to substitute the *A*-site La^3+^ and *B*-site Sc^3+^, respectively. Interestingly, improvements in the ionic conductivity of (Li*_x_*La_1−*x*/3_)ScO_3_ were achieved only upon Ce^4+^ co-doping, with the presence of Zr^4+^ and Nb^5+^ having no such effect. A mono-phasic sample was obtained for the co-doped (Li_0.45_La_0.78_Ce_0.05_)ScO_3_ system, which exhibited a higher ionic conductivity compared to (Li_0.45_La_0.85_)ScO_3_. Furthermore, an increase in the activation energy indicated that a rise in the pre-exponential factor (σ_0_) might contribute to the enhanced conductivity. Compositional analysis and crystal structure refinement revealed the real compositions of (Li_0.45_La_0.85_)ScO_3_ and (Li_0.45_La_0.78_Ce_0.05_)ScO_3_ to be Li_0.186_La_0.938_ScO_3_ and Li_0.493_La_0.777_Ce_0.044_ScO_3_, respectively. Our results also indicated that an increase in the lithium amount and expansion of the unit cell volume could contribute to the higher ionic conductivity of (Li_0.45_La_0.78_Ce_0.05_)ScO_3_ (i.e., 1.9 × 10^−4^ S cm^−1^ at 623 K). Indeed, even a small amount of Ce^4+^ co-doping had a sufficient effect on the ionic conductivity since it can significantly modify the number of charge carriers in the developed (Li*_x_*La_1−*x*/3_)ScO_3_-based materials. Although the ionic conductivity of the obtained materials in this study is not yet very high, these materials are among the rarely studied lithium ion conductors with a perovskite structure consisting of a single-valent metal element at the *B*-site. Moreover, the lower valency of Sc^3+^ at *B*-site suggested that greater number of lithium interstitials could be generated compared to in the case of Ti^4+^ at the *B*-site, thereby rendering a higher carrier doping possibility. The obtained materials could be considered for use as template materials to further improve ionic conductivities until they have been further developed to approach a high lithium ion conductivity at low temperatures for practical use. These results are expected to contribute to the development of superior lithium-based solid electrolytes for application in all-solid-state lithium batteries, and the ongoing material search will be expected to expand the varieties of materials available for use in battery applications.

## Figures and Tables

**Table 1:** Ionic conductivities at 623 K and activation energies of the obtained (Li*_x_*La_1−*x*/3_)ScO_3_.

Formula	Nominal Composition	Identified Phases	σ_total_/S cm^−1^	*E*_a_/kJ mol^−1^
(Li*_x_*La_1−*x*/3_)ScO_3_	LaScO_3_	LaScO_3_-type	−	−
	(Li_0.15_La_0.95_)ScO_3_	LaScO_3_-type	3.4 × 10^−7^	143.0 ± 6.8
	(Li_0.30_La_0.90_)ScO_3_	LaScO_3_-type	2.8 × 10^−6^	91.6 ± 1.7
	(Li_0.45_La_0.85_)ScO_3_	LaScO_3_-type	4.2 × 10^−5^	61.0 ± 0.9
	(Li_0.60_La_0.80_)ScO_3_	LaScO_3_-type, LaLiO_2_	7.2 × 10^−6^	80.7 ± 2.2

**Table 2:** Dependence of the ionic conductivities at 623 K and the activation energies on the Ce^4+^, Zr^4+^, and Nb^5+^ co-doped (Li*_x_*La_1−*x*/3_)ScO_3_ compositions.

Chemical Formula	Composition	Identified Phases	σ_total_/S cm^−1^	*E*_a_/kJ mol^−1^
(Li*_x_*La_1−*x*/3_)ScO_3_	(Li_0.45_La_0.85_)ScO_3_	LaScO_3_-type	4.2 × 10^−5^	61.0 ± 0.9
(Li*_x_*La_1−*x*/3−4*y*/3_Ce*_y_*)ScO_3_	*x* = 0.35 (fixed), *y* = 0.05 (Li_0.35_La_0.82_Ce_0.05_)ScO_3_	LaScO_3_-type, CeO_2_, unknown	2.1 × 10^−5^	88.9 ± 0.7
	*x* = 0.35 (fixed), *y* = 0.10 (Li_0.35_La_0.75_Ce_0.10_)ScO_3_	LaScO_3_-type, CeO_2_	2.4 × 10^−5^	87.9 ± 0.3
	*x* = 0.40 (fixed), *y* = 0.10 (Li_0.40_La_0.73_Ce_0.10_)ScO_3_	LaScO_3_-type, CeO_2_	8.8 × 10^−4^	75.5 ± 0.6
	*x* = 0.40 (fixed), *y* = 0.15 (Li_0.40_La_0.67_Ce_0.15_)ScO_3_	LaScO_3_-type, CeO_2_	1.1 × 10^−3^	75.4 ± 0.1
	*x* = 0.40, *y* = 0.05 (fixed) (Li_0.50_La_0.77_Ce_0.05_)ScO_3_	LaScO_3_-type, CeO_2_	5.5 × 10^−5^	78.2 ± 2.5
	*x* = 0.45, *y* = 0.05 (fixed) (Li_0.45_La_0.78_Ce_0.05_)ScO_3_	LaScO_3_-type	1.9 × 10^−4^	82.9 ± 2.1
	*x* = 0.50, *y* = 0.05 (fixed) (Li_0.50_La_0.72_Ce_0.10_)ScO_3_	LaScO_3_-type, CeO_2_, unknown	1.3 × 10^−5^	86.5 ± 0.3
(Li_0.45_La_0.85−0.33*z*_)(Sc_1−*z*_Zr*_z_*)O_3_	(Li_0.45_La_0.833_)(Sc_0.95_Zr_0.05_)O_3_	LaScO_3_-type, LiLaO_2_	7.36 × 10^−6^	77.9 ± 2.0
	(Li_0.45_La_0.817_)(Sc_0.90_Zr_0.10_)O_3_	LaScO_3_-type, LiLaO_2_	8.03× 10^−6^	75.6 ± 1.8
(Li_0.45_La_0.85−0.67*z*_)(Sc_1−*z*_Nb*_z_*)O_3_	(Li_0.45_La_0.817_)(Sc_0.95_Nb_0.05_)O_3_	LaScO_3_-type, LiLaO_2_	3.67 × 10^−6^	81.1 ± 0.9
	(Li_0.45_La_0.783_)(Sc_0.90_Nb_0.10_)O_3_	LaScO_3_-type, LiLaO_2_	9.50 × 10^−6^	82.0 ± 1.9

**Table 3:** Crystallographic parameters of the synchrotron diffraction data for (Li_0.45_La_0.85_)ScO_3_ refined by Rietveld analysis.

Atom	Site	*g*	*x*	*y*	*z*	*B*_iso_/Å^2^
La	4*c*	0.938(2)	0.95637(10)	0.25	0.01062(13)	0.235(9)
Li	4*c*	=1–*g*(La)	=*x* (La)	=*y* (La)	=*z* (La)	=*B* (La)
Sc	4*b*	1.0	0.5	0	0	0.908(7)
O1	4*c*	1.0	0.53871(1)	0.25	0.90136(4)	0.788(6)
O2	8*d*	1.0	0.29065(7)	0.94553(6)	0.70253(7)	0.848 (3)

*a* = 5.67921(5) Å, *b* = 5.79488(4) Å and *c* = 8.09558(6) Å, *V* = 266.4281(36); *R*_wp_ = 8.638, *S* = 1.899, *R*_b_ = 6.080, *R*_f_ = 4.965.

**Table 4:** Crystallographic parameters of the synchrotron diffraction data for (Li_0.45_La_0.78_Ce_0.05_)ScO_3_ refined by Rietveld analysis

Atom	Site	*g*	*x*	*y*	*z*	*B*_iso_/ Å^2^
La	4*c*	0.8212(5)	0.95622(4)	0.25	0.00892(6)	0.178(3)
Li	4*c*	=1–*g*(La)	=*x* (La)	=*y* (La)	=*z* (La)	=*B* (La)
Sc	4*b*	1.0	0.5	0	0	1.209(4)
O1	4*c*	1.0	0.53153(4)	0.25	0.90757(5)	0.699(3)
O2	8*d*	1.0	0.2951(6)	0.9419(3)	0.70075(3)	1.825(4)

*a*= 5.68088(6) Å, *b* = 5.79624(6) Å and *c* = 8.09803(9) Å, *V* = 266.6500(48); *R*_wp_ = 8.850, *S* = 2.315, *R*_b_ = 5.343, *R*_f_ = 3.752.

## Data Availability

The data presented in this study are available in supplementary material.

## Supplementary Materials

The following are available online. Figure S1: (a) X-ray diffraction patterns, (b) observed shifts of the selected reflections, and (c) variation in the lattice parameters with the composition of (Li_0.45_La_0.85−0.33*z*_)(Sc_1−*z*_Zr*_z_*)O_3_ (*y* = 0 and 0.1). Figure S2: (a) X-ray diffraction patterns, (b) observed shifts of the selected reflections, and (c) variation in the lattice parameters with the composition of (Li_0.45_La_0.85−0.67*z*_)(Sc_1−*z*_Nb*_z_*)O_3_ (*z* = 0 and 0.1). Figure S3: Temperature dependence of the sample conductivity in (a) (Li_0.45_La_0.85−*z*/3_)(Sc_1−*z*_Zr*_z_*)O_3_ and (Li_0.45_La_0.83−1.33*n*_)(Sc_0.95_Zr_0.05_)O_3_, and (b) (Li_0.45_La_0.85−2*z*/3_)(Sc_1−*z*_Nb*_z_*)O_3_. Figure S4: Steady-state current as a function of the applied voltage at 623 K in a dry Ar atmosphere for (Li_0.4_La_0.67_Ce_0.15_)ScO_3_. Table S1: Chemical compositions of (Li_0.45_La_0.85_)ScO_3_ and (Li_0.45_La_0.78_Ce_0.05_)ScO_3_.

## Sample Availability

Not available.
