# Supplementary material for: Syntheses and Characterization of Novel Perovskite-Type LaScO3-Based Lithium Ionic Conductors
Source: Molecules. 2021 Jan 8;26(2):299. doi: 10.3390/molecules26020299 (PMC7828101; doi:10.3390/molecules26020299)
Supplement: Supplementary file 1 [file molecules-26-00299-s001.pdf]

molecules

MDPI

Supporting Information

High-pressure syntheses, structures, and ionic conductivities of perovskite-type $LaScO_3$-based lithium ionic conductors

Guowei Zhao, [a] Kota Suzuki, [b, c] Masaaki Hirayama, [b] Ryoji Kanno *[a] and [b]

[a] All-Solid-State Battery Unit, Institute of Innovation Research, Tokyo Institute of Technology, 4259 Nagatsuta, Midori-ku, Yokohama 226-8502, Japan; zhao.g.w@echem.titech.ac.jp

[b] Department of Chemical Science and Engineering, School of Materials and Chemical Technology, Tokyo Institute of Technology, 4259 Nagatsuta, Midori-ku, Yokohama 226-8502, Japan; suzuki.k.bf@m.titech.ac.jp (K.S.); hirayama@echem.titech.ac.jp (M.H.);

[c] Precursory Research for Embryonic Science and Technology (PRESTO), Japan Science and Technology Agency (JST), 4-1-8 Honcho Kawaguchi-shi, Saitama, 332-0012, Japan;

* Corresponding Author, E-mail: kanno@echem.titech.ac.jp.

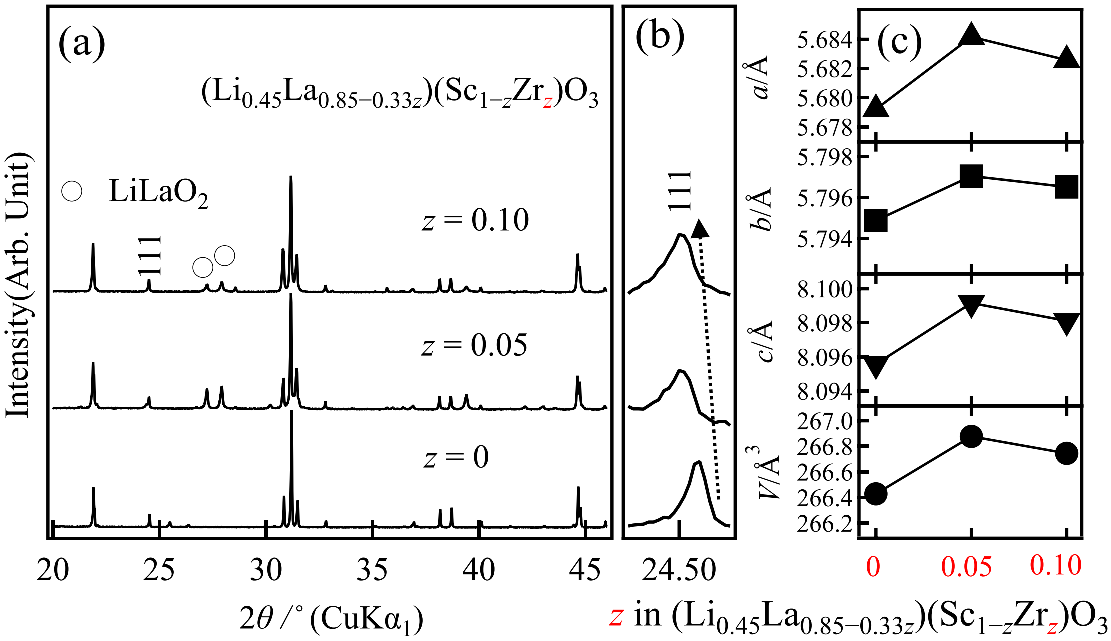

Figure 1. (**a**) X-ray diffraction patterns, (**b**) observed shifts of the selected reflections, and (**c**) variation in the lattice parameters with the composition of $(Li_{0.45}La_{0.85-0.33z})(Sc_{1-z}Zr_z)O_3$ (y = 0 and 0.1).

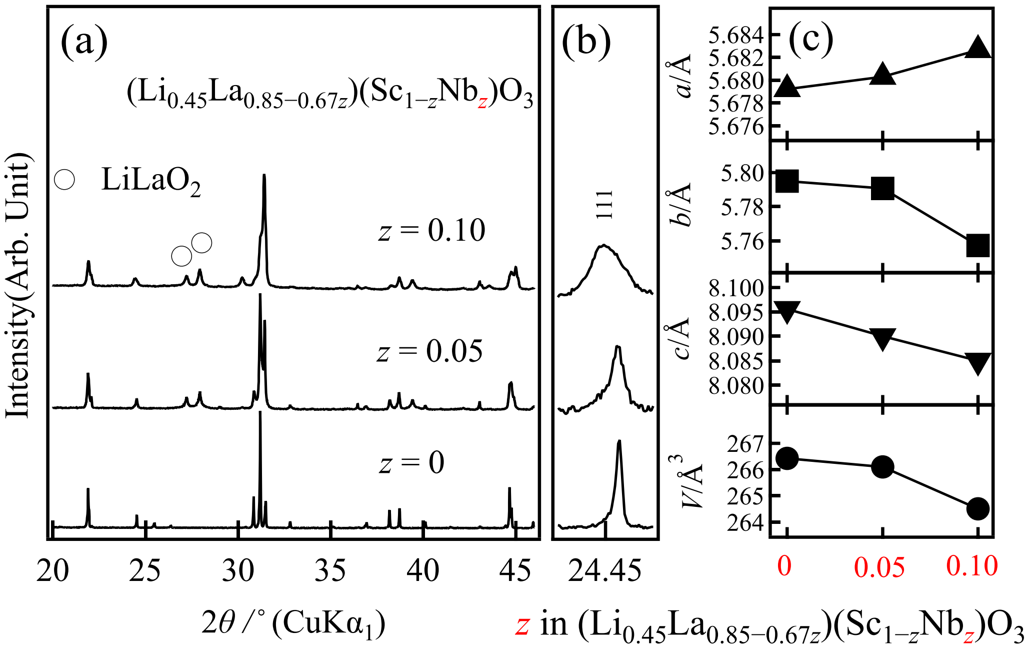

Figure 2. (**a**) X-ray diffraction patterns, (**b**) observed shifts of the selected reflections, and (**c**) variation in the lattice parameters with the composition of $(Li_{0.45}La_{0.85-0.67z})(Sc_{1-z}Nb_z)O_3$ (z = 0 and 0.1).

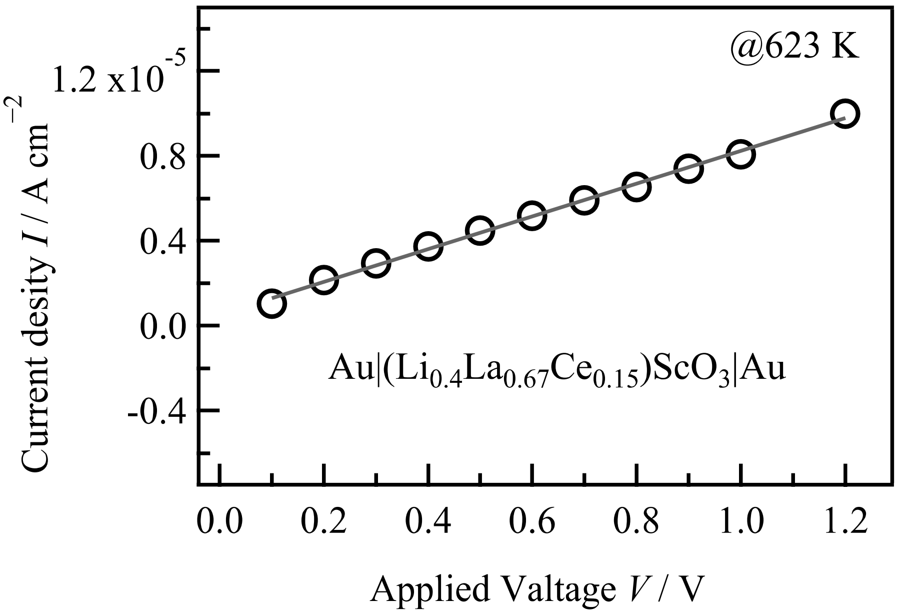

Figure 3. Steady-state current as a function of the applied voltage at 623 K in dry Ar atmosphere for ($Li_{0.4}La_{0.67}Ce_{0.15}$)$ScO_3$.

The chemical compositions of ($Li_{0.45}La_{0.85}$)ScO_3 and ($Li_{0.45}La_{0.78}Ce_{0.05}$)$ScO_3$ (Table 1), determined by inductively coupled plasma atomic emission spectroscopy (ICP-AES: Shimadzu, ICPS-8100), were comparable to the nominal values.

Table 1. Chemical compositions of ($Li_{0.45}La_{0.85}$)ScO_3 and ($Li_{0.45}La_{0.78}Ce_{0.05}$)$ScO_3$.

	**Li**	**La**	**Ce**	**Sc**
Nominal	0.45	0.85	–	1
ICP-AES	0.193(2)	0.924(4)	–	0.954(3)
Nominal	0.45	0.78	0.05	1
ICP-AES	0.463(4)	0.746(3)	0.042(5)	0.983(2)
